# Expression Pattern and Prognostic Significance of the Long Non-Coding RNA Metastasis-Associated Lung Adenocarcinoma Transcript 1 in Chronic Lymphocytic Leukemia

**DOI:** 10.3390/ijms25020922

**Published:** 2024-01-11

**Authors:** Kristina Tomic Vujovic, Milena Ugrin, Natasa Tosic, Vojin Vukovic, Irena Marjanovic, Tatjana Kostic, Sanja Stankovic, Vladimir Otasevic, Sofija Sarac, Darko Antic, Sonja Pavlovic, Teodora Karan-Djurasevic

**Affiliations:** 1 Clinic for Hematology, University Clinical Center of Serbia, 11000 Belgrade, Serbia; kristinat992@gmail.com (K.T.V.); vojinvukovic@yahoo.com (V.V.); vladimirota@hotmail.com (V.O.); sofijasarac@gmail.com (S.S.); darkoantic1510976@gmail.com (D.A.); 2 Institute of Molecular Genetics and Genetic Engineering, University of Belgrade, 11042 Belgrade, Serbia; milenaradmilovic@gmail.com (M.U.); natasa.tosic@imgge.bg.ac.rs (N.T.); irena.marjanovic@imgge.bg.ac.rs (I.M.); tanjaburlo@gmail.com (T.K.); sonja.pavlovic99@gmail.com (S.P.); 3 Center for Medical Biochemistry, University Clinical Center of Serbia, 11000 Belgrade, Serbia; sanjast2013@gmail.com; 4 Department of Biochemistry, Faculty of Medical Sciences, University of Kragujevac, 34000 Kragujevac, Serbia; 5 School of Medicine, University of Belgrade, 11000 Belgrade, Serbia

**Keywords:** long non-coding RNA, MALAT1, chronic lymphocytic leukemia, expression, prognosis

## Abstract

Dysregulated expression of the long non-coding RNA MALAT1 has been implicated in the pathogenesis and progression of a variety of cancers, including hematological malignancies, but it has been poorly investigated in chronic lymphocytic leukemia (CLL). In this study, the expression of MALAT1 was measured using a quantitative reverse-transcriptase polymerase chain reaction in the peripheral blood mononuclear cells of 114 unselected, newly diagnosed CLL patients in order to analyze its association with clinical, laboratory, and molecular patients’ characteristics at diagnosis, as well as its prognostic relevance. MALAT1 was found to be upregulated in CLL patients in comparison to healthy controls, and expression levels were not related to age, leukocyte, lymphocyte and platelet count, serum β2-microglobulin, and *IGHV* somatic hypermutational status. On the other hand, high MALAT1 expression was associated with several favorable prognostic markers (high hemoglobin, low serum lactate dehydrogenase, earlier clinical stages, CD38-negative status), but also with unfavorable cytogenetics. Furthermore, an association between high MALAT1 levels and longer time to first treatment and overall survival in *IGHV*-unmutated CLL subtype was observed. In summary, our results imply that high MALAT1 expression at diagnosis may be a predictor of better prognosis and point to MALAT1 expression profiling as a candidate biomarker potentially useful in clinical practice.

## 1. Introduction

Long non-coding RNAs (lncRNAs) belong to a heterogeneous class of transcripts without a protein-coding function and are defined by their length being greater than 200 nucleotides. LncRNAs are transcribed from intergenic regions and regions partially overlapping coding, intronic, or regulatory sequences of protein-coding genes on the sense or antisense strand. The majority of lncRNAs are transcribed by RNA polymerase II and, like mRNAs, undergo 5′-end capping, 3′-end polyadenylation, and splicing. They are predominantly located in the nucleus but are also exported to cytosol and organelles (mitochondria and exosomes). Through interactions with DNA, RNA, and proteins, lncRNAs regulate gene expression in cis and in trans at the epigenetic, transcriptional, post-transcriptional, translational, and post-translational level. Consequently, lncRNAs participate in the fine-tuning of multiple cellular processes in a cell-type-dependent and context-dependent manner and have been implicated in the pathogenesis and progression of many diseases, including cancer [1,2].

MALAT1 (metastasis-associated lung adenocarcinoma transcript 1; also known as NEAT2, nuclear-enriched abundant transcript 2) was the first identified and best characterized lncRNA, whose role in the initiation and clinical behavior of different types of cancer has been widely studied. It exhibits several characteristics untypical for lncRNAs, such as high sequence conservation, ubiquitous expression, and high abundance across all cell types [3,4,5]. MALAT1 is an 8.7 kb long transcript encoded by a single-exon gene located on chromosome 11q13.1. Primary MALAT1 transcript is processed by the cleavage of a tRNA-like small RNA from its 3′-end, followed by the formation of a 3′-end triple helical structure which stabilizes the resulting mature MALAT1 lncRNA [6]. Increased stability and a high level of transcription are the main causes of MALAT1 RNA abundance in cells. The 3′-end triple helix, along with other domains, is responsible for the retention of MALAT1 in the nucleus within the nuclear speckles, nuclear condensates that accumulate splicing factors, as well as RNA processing and export factors [6,7,8,9,10,11]. MALAT1 is not involved in the formation of nuclear speckles but is being recruited to the speckle periphery via interactions with their protein components upon the initiation of transcription [7,8,11]. In nuclear speckles, MALAT1 regulates alternative splicing through direct interaction with serine/arginine-rich splicing factors (SRSFs), modulating their phosphorylation and leading to the recruitment of SRSFs to the site of transcription and pre-mRNA [11]. In addition to localization in nuclear speckles, MALAT1 is also associated with chromatin at actively transcribed loci [12,13]. It is involved in gene regulation by: (1) interactions with transcription factors and transcriptional co-activators (e.g., Sp1, FOXO1, p300) recruiting them to the promoters of target genes, and (2) interactions with the components of polycomb epigenetic–regulatory complexes (e.g., Pc2, EZH2, SUZ12) [14,15,16,17,18,19,20]. Through miRNA-responsive elements in its sequence, MALAT1 also acts as competitive endogenous RNA (ceRNA) which sponges miRNAs (e.g., miR-195, miR-125b, miR-376a, miR-22), thus reducing the inhibitory effect of miRNAs on their target mRNAs [21,22,23,24]. Employing various mechanisms of action, MALAT1 modulates multiple cellular pathways (such as MAPK/ERK, PI3K/AKT, β-catenin/Wnt, Hippo-YAP, VEGF, NF-κB, etc.) which regulate proliferation, apoptosis, communication with the microenvironment, and other cancer-related processes [25].

The upregulation of MALAT1 has been observed in many solid tumors and hematological malignancies, and high expression was found to be associated with the progression of disease, chemotherapy resistance, metastatic potential, and reduced patients’ survival, implying its pro-oncogenic role [3,26,27,28,29]. However, other studies have reported decreased MALAT1 expression in some types of cancers, as well as the association of low MALAT1 levels with advanced clinical stages and metastases. These findings have pointed to the tumor-suppressive effects of MALAT1, causing a controversy that remains to be resolved [3,28,29]. 

Chronic lymphocytic leukemia (CLL) is a malignancy of mature CD5^+^ B lymphocytes which affects mainly elderly individuals (median age at diagnosis is 72 years), predominantly men [30]. Although commonly considered to be an indolent disease that can persist as stable lymphocytosis for a long period of time without the need for treatment, CLL can also follow a rapidly progressive course, requiring therapy soon after diagnosis and with short overall survival (OS). The complex pathobiology of CLL cells, which underlies the clinical heterogeneity of the disease, results from their intrinsic genetic alterations and crosstalk with the microenvironment. In particular, (auto)antigenic stimulation and signaling through the B-cell receptor (BCR) play a critical role in the pathogenesis and progression of CLL, which is evidenced by the strong prognostic and predictive relevance of the BCR composition (i.e., somatic hypermutational status of immunoglobulin heavy-variable (*IGHV*) genes and BCR stereotypy) [31,32]. Besides recurrent chromosomal aberrations (del13q14, trisomy 12, del11q22-23, del17p13) and mutations of protein-coding genes (*NOTCH1*, *SF3B1*, *ATM*, *TP53*, *BIRC3*, etc.), alterations of non-coding RNAs have also been detected in CLL. Regarding lncRNAs, the aberrant expression of a number of them has been reported (e.g., MIAT, LincRNA-p21, NEAT1, CRNDE, TRERNA1, lnc-TOMM7-1, GAS5, BM742401, etc.), as well as their effect on different aspects of CLL cells’ biology. Some of the deregulated lncRNAs exerted an association with the established prognostic markers in CLL, the disease course, and outcome [33,34,35,36,37]. On the other hand, lncRNA MALAT1 has been extremely under-researched in CLL. There are only a few studies that investigated MALAT1 expression in CLL, in which MALAT1 upregulation was detected [38,39]. While our research was being conducted, Fernandez-Garnacho et al. reported the association of MALAT1 overexpression with aggressive behavior of CLL. The authors observed an adverse prognostic impact of high MALAT1 levels on the time to first treatment (TTFT), and a positive correlation between the expression of MALAT1 and genes involved in pathways transducing signals from the lymph node microenvironment [39].

Here, we present the results of our study in which we investigated the association of lncRNA MALAT1 expression with the clinico-biological characteristics of CLL patients at diagnosis, and its potential prognostic significance.

## 2. Results

### 2.1. Description of the Study Cohort

This study included a total of 114 CLL patients and 20 healthy controls. The patient group consisted of 81 men and 33 women (male/female = 2.45:1), aged 33−80 years (median = 59) at diagnosis. The control group consisted of 15 men and 5 women (male/female = 3:1), with a median age of 71 years (range: 65−85). Clinical and molecular characteristics of the CLL cohort are depicted in Table 1.

The median follow-up time of the CLL patients was 73.5 months (range: 4−360), during which 60 patients (52.6%) died, 44 patients (38.6%) were still alive, while in 10 patients, (8.8%) their outcome was unknown. Median overall survival (OS) was 92 months (95% confidence interval (CI) = 75.6−108.4). During the follow-up period, 90 patients (78.9%) underwent treatment, while 24 patients (21.1%) remained untreated. TTFT ranged from 0 to 348 months, with the median of 22 months (95% confidence interval (CI) = 8.4−35.6). 

### 2.2. LncRNA MALAT1 Expression and the Relationship with the Disease-Related Variables

**Figure 1:**
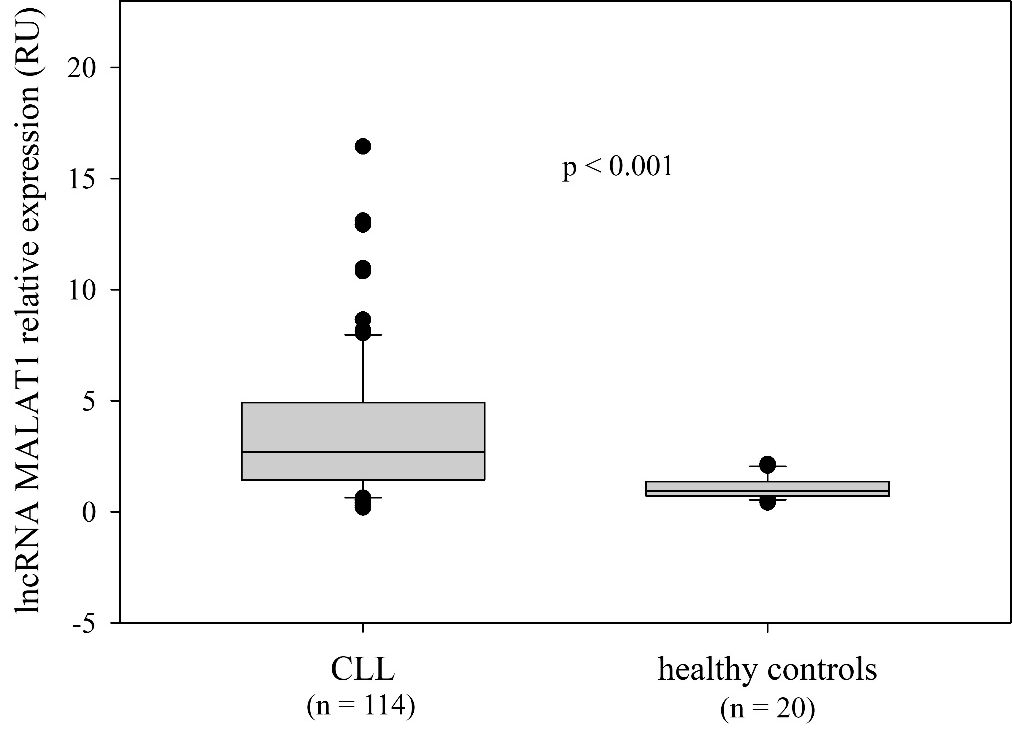
MALAT1 relative expression in mononuclear cells of CLL patients vs. healthy controls. Quantification of MALAT1 by qRT-PCR showed a significantly higher expression in CLL samples in comparison to non-leukemic samples (*p* < 0.001; Mann–Whitney rank-sum test). CLL: median 2.7 RU; range 0.19−67.51 RU; 25th percentile 1.445 RU; 75th percentile 4.915 RU. Healthy controls: median 0.94 RU; range 0.41−2.15 RU; 25th percentile 0.71 RU; 75th percentile 1.368 RU. qRT-PCR, quantitative reverse-transcriptase polymerase chain reaction; RU, relative units; *n*, number of patients in the analyzed group.

The analysis of MALAT1 expression in peripheral blood mononuclear cells using a quantitative reverse-transcriptase polymerase chain reaction (qRT-PCR) revealed significantly higher levels of MALAT1 transcripts in CLL patients in comparison to healthy controls (*p* < 0.001, Mann–Whitney rank-sum test). While MALAT1 expression showed little variation across the control group, it was highly variable among CLL patients (Figure 1). For the purpose of further analyses, a median value of MALAT1 relative expression (2.7 relative units (RU)) was used as a cut-off to dichotomize the CLL cohort into MALAT1 low-expressing (MALAT1^low^) and MALAT1 high-expressing (MALAT1^high^) groups. The median MALAT1 expression in the MALAT1^low^ group was 1.45 RU (range 0.19−2.69; 25th percentile 0.705 RU; 75th percentile 2.1 RU), whereas in the MALAT1^high^ group it was 4.9 RU (range 2.71−67.51; 25th percentile 3.765 RU; 75th percentile 7.495 RU). The comparison of these two groups regarding the clinical and biological characteristics of patients is presented in Table 1.

In both CLL and the healthy control group, the expression of MALAT1 was not associated with age. Since the median age of our CLL patients was lower than in the general CLL population, as well as in the control group, we also investigated the association of MALAT1 expression with age at diagnosis in two age subgroups: ˂65 and ≥65 years. MALAT1 levels were higher in older patients than in younger patients, but this difference was not statistically significant (*p* = 0.182, Mann–Whitney rank-sum test). In both groups, MALAT1 expression was not associated with age at diagnosis and was significantly higher than in the control group (*p* < 0.001, Mann–Whitney rank-sum test). Regarding the male/female ratios, the composition of CLL and healthy control cohorts was comparable. In the CLL group, the expression of MALAT1 was higher in male than in female patients (*p* = 0.011, Mann–Whitney rank-sum test), in contrast to the control group, where higher MALAT1 expression was observed in female patients (*p* = 0.022, Mann–Whitney rank-sum test).

When analyzing the association of MALAT1 expression with the baseline disease-related variables, no significant differences between the MALAT1^low^ and MALAT1^high^ group concerning white blood cell (WBC), lymphocyte and platelet counts, and β2-microglobulin levels were detected. On the other hand, there was a positive correlation between MALAT1 expression and hemoglobin levels (*p* = 0.001, r = 0.326, Spearman rank-order correlation test); MALAT1^high^ patients had significantly higher hemoglobin levels in comparison to MALAT1^low^ patients (*p* = 0.004, Mann–Whitney rank-sum test). In addition, a negative correlation between lactate dehydrogenase (LDH) levels and MALAT1 expression was observed (*p* = 0.002, r = −0.354, Spearman rank-order correlation test); median LDH levels were significantly lower in the MALAT1^high^ vs. MALAT1^low^ group (*p* = 0.004, Mann–Whitney rank-sum test). 

**Figure 2:**
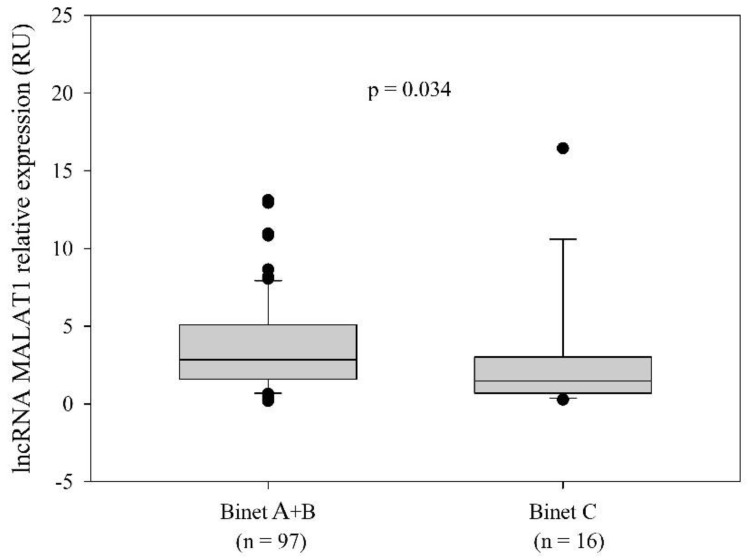
Association of MALAT1 relative expression with Binet clinical stages at diagnosis. qRT-PCR analysis revealed higher MALAT1 expression in the group of CLL patients consisting of Binet A and Binet B cases than in the group of Binet C cases (*p* = 0.034; Mann–Whitney rank-sum test). Binet A + B: median 2.84 RU; range 0.19−67.51 RU; 25th percentile 1.595 RU; 75th percentile 5.07 RU. Binet C: median 1.475 RU; range 0.27−16.44 RU; 25th percentile 0.698 RU; 75th percentile 3.017 RU.

The MALAT1^high^ and MALAT1^low^ groups were not different in terms of the distribution of clinical Binet stages at diagnosis (*p* = 0.250; χ^2^ test). However, when patients at low-risk Binet A and intermediate-risk Binet B stages were grouped together (Binet A + B), a significantly higher MALAT1 expression was detected in the Binet A + B group compared to patients at the high-risk Binet C stage (*p* = 0.034, Mann–Whitney rank-sum test) (Figure 2).

**Figure 3:**
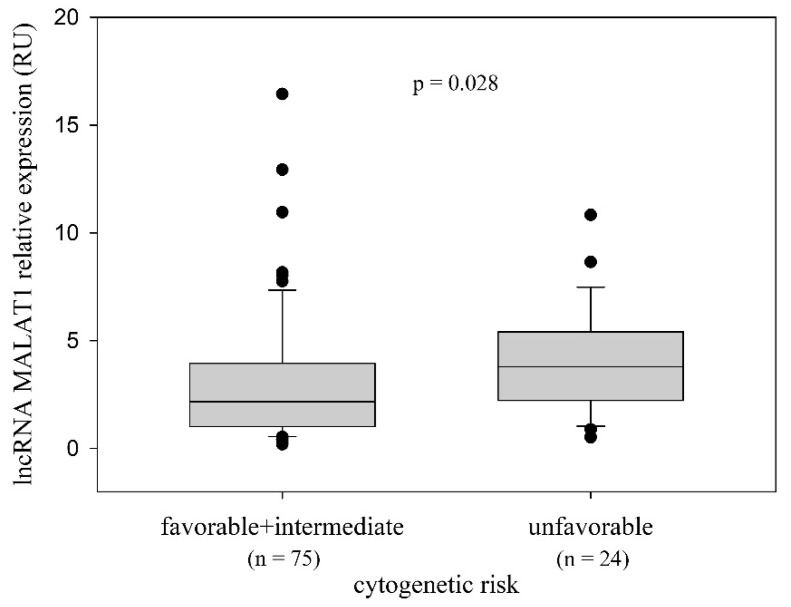
Association of MALAT1 relative expression with cytogenetic risk groups. qRT-PCR analysis revealed higher MALAT1 expression in the group of CLL patients consisting of cases with unfavorable cytogenetic risk than in the group combining patients with favorable and intermediate cytogenetic risk (*p* = 0.028; Mann–Whitney rank-sum test). Favorable and intermediate cytogenetic risk: median 2.17 RU; range 0.19−16.44 RU; 25th percentile 1.02 RU; 75th percentile 3.95 RU. Unfavorable cytogenetic risk: median 3.79 RU; range 0.51−10.83 RU; 25th percentile 2.228 RU; 75th percentile 5.41 RU.

Similarly to Binet stages, the frequencies of cytogenetic risk groups (favorable—del13q as a sole chromosomal abnormality; intermediate—no aberrations, trisomy 12q; unfavorable—del11q, del17p) did not differ in the MALAT1^high^ and MALAT1^low^ groups (*p* = 0.133; χ^2^ test). However, when we grouped cases with favorable and intermediate cytogenetic risk together, we observed a higher expression of MALAT1 in patients with unfavorable cytogenetic risk than in patients with favorable and intermediate cytogenetic risk (*p* = 0.028, Mann–Whitney rank-sum test) (Figure 3). 

**Figure 4:**
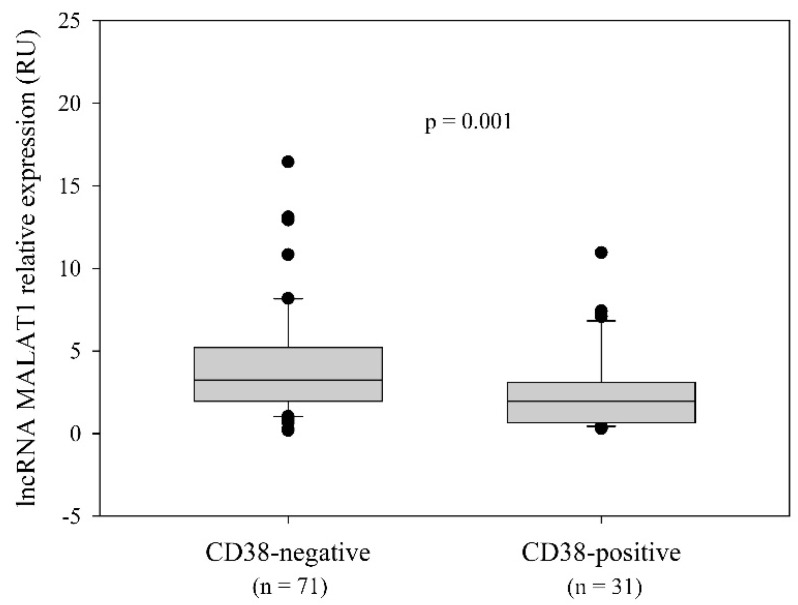
Association of MALAT1 relative expression with CD38 status. qRT-PCR analysis revealed higher MALAT1 expression in CD38-negative vs. CD38-positive patients (*p* = 0.001; Mann–Whitney rank-sum test). CD38-negative: median 3.24 RU; range 0.19−67.51 RU; 25th percentile 1.95 RU; 75th percentile 5.2 RU. CD38-positive: median 1.95 RU; range 0.3−10.96 RU; 25th percentile 0.64 RU; 75th percentile 3.09 RU.

We also investigated the association of MALAT1 expression and surface CD38 expression and observed higher MALAT1 levels in CD38-negative vs. CD38-positive patients (*p* = 0.001, Mann–Whitney rank-sum test) (Figure 4). 

Association of MALAT1 expression with CLL subtypes defined by *IGHV* somatic hypermutational (SHM) status (*IGHV*-mutated, M-CLL, and *IGHV*-unmutated, U-CLL) was not detected (*p* = 1.000, Fisher exact test). The analysis of patient-specific *IGHV-IGHD-IGHJ* rearrangements revealed BCR stereotypy in 15 patients (13.2%). All of the detected stereotyped BCRs belonged to the “major” stereotyped subsets and were predominantly present in the U-CLL subtype (13/15 patients, 86.7%). Among U-CLL cases, the identified stereotyped BCRs were assigned to subset #1 (5 patients), subset #3 (3 patients), subset #5 (2 patients), subset #31 (1 patient), subset #99 (1 patient), and subset #202 (1 patient). Among M-CLL cases, subset #2 (1 patient) and subset #4 (1 patient) were detected. We then investigated the association of MALAT1 expression with BCR stereotypy and observed no significant difference regarding MALAT1 levels between stereotyped and non-stereotyped cases (*p* = 0.291, Mann–Whitney rank-sum test). The same lack of association was also observed in the U-CLL subtype (*p* = 0.318, Mann–Whitney rank-sum test) and in the M-CLL subtype (*p* = 0.493, Mann–Whitney rank-sum test). Three out of six patients assigned to subset #1 and its satellite subset #99, both of which are associated with the adverse prognosis, which belonged to the MALAT1^high^ group, while the other three patients belonged to the MALAT1^low^ group. The patient with subset #2 BCR, which is considered to be a marker of poor prognosis regardless of *IGHV* SHM status, and the patient with subset #4, associated with indolent clinical course, both expressed high MALAT1 levels.

We have also analyzed the association between *TP53* mutational status and MALAT1 expression in 61 patients for whom we possessed this information and found that MALAT1 levels were not significantly different in *TP53*-wild type cases and *TP53*-mutated cases (*p* = 0.536, Mann–Whitney rank-sum test).

The International Prognostic Index for CLL (CLL-IPI) was calculated for 55 patients for whom we possessed complete data (age at diagnosis, Binet stage, serum β2-microglobulin, *IGHV* mutational status, and *TP53* aberrations (mutations of *TP53* and/or del17p)). We observed no differences in the distribution of the CLL-IPI risk groups (low, intermediate, high, very high) between MALAT1^low^ and MALAT1^high^ patients either in the whole cohort (*p* = 0.228, Fisher exact test) or in the M-CLL and the U-CLL subtype separately (*p* = 0.706 and *p* = 0.42, Fisher exact test, respectively). When we grouped patients with low and intermediate and high and very high risk together, we observed a trend towards a higher frequency of high and very high CLL-IPI risk in the MALAT1^high^ group in comparison to the MALAT1^low^ group when the whole cohort was analyzed, but this difference did not reach statistical significance (*p* = 0.08, Fisher exact test). In M-CLL and U-CLL, this difference was also not significant (*p* = 0.557 and *p* = 0.175, Fisher exact test, respectively). It should be noted, though, that in 16 out of 55 patients, the CLL-IPI scores were calculated without information about *TP53* mutational status.

### 2.3. LncRNA MALAT1 Expression and Clinical Outcomes

**Figure 5:**
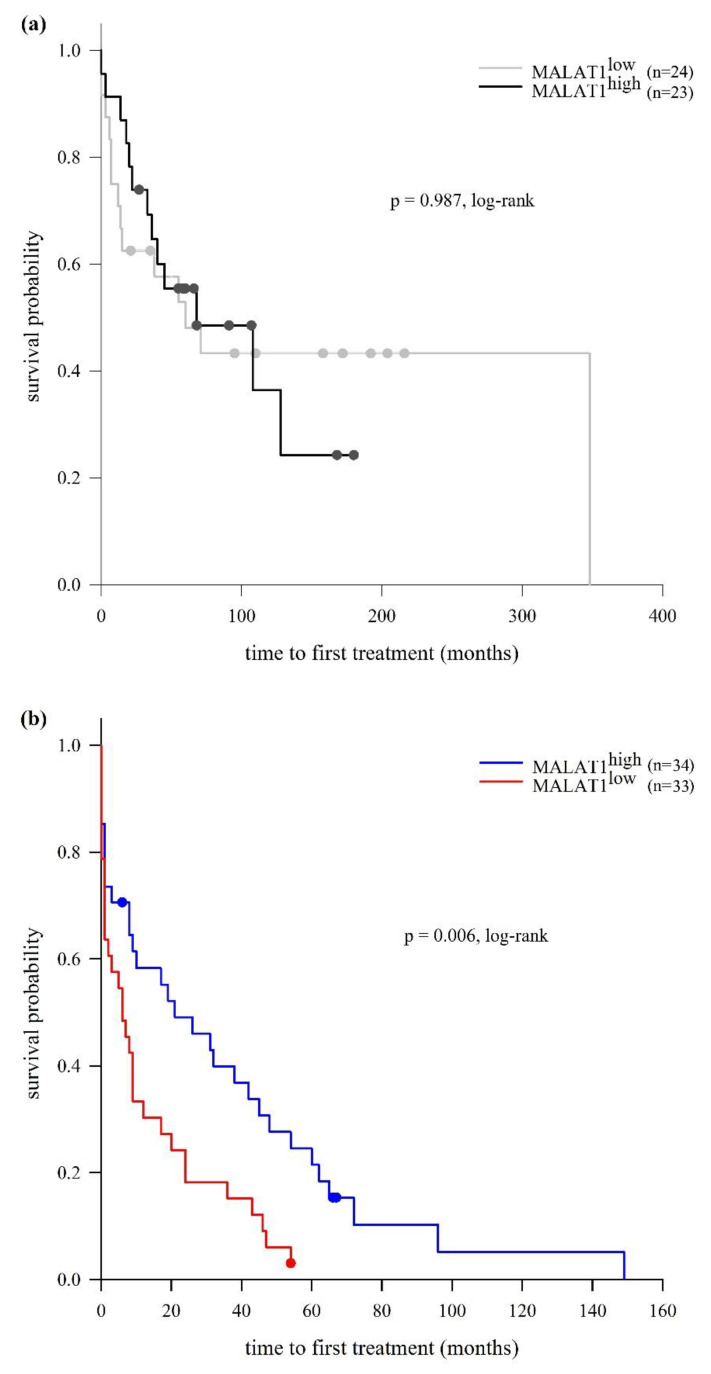
Kaplan–Meier analysis of the time to first treatment (TTFT) in relation to MALAT1 expression in M-CLL (**a**) and U-CLL (**b**). Significant differences in TTFT between MALAT1^high^ and MALAT1^low^ group were observed in the U-CLL, but not in the M-CLL subtype. (**a**) M-CLL (*n* = 47): median TTFT in MALAT1^high^ group was 68 months (95% CI = 5.063−141.063) and in MALAT1^low^ group 60 months (95% CI = 12.727−107.203). (**b**) U-CLL (*n* = 67): median TTFT in MALAT1^high^ group was 21 months (95% CI = 1.589−40.411) and in MALAT1^low^ group 6 months (95% CI = 0.373−11.627). CI, confidence interval.

The analysis of TTFT in our CLL cohort revealed longer TTFT in the MALAT1^high^ group in comparison to the MALAT1^low^ group (38 and 12 months, respectively), but this difference was not statistically significant (*p* = 0.273, log-rank test). We then investigated the relationship between MALAT1 expression and TTFT in M-CLL and U-CLL subtypes separately. This approach showed that, while in the M-CLL subtype the MALAT1^high^ and MALAT1^low^ groups were very similar in terms of the median duration of the period before the initiation of treatment (68 and 60 months, respectively; *p* = 0.987, log-rank test), in the U-CLL subtype, TTFT was significantly longer in the MALAT1^high^ vs. MALAT1^low^ group (21 vs. 6 months, respectively; *p* = 0.006, log-rank test) (Figure 5). Multivariate Cox regression analysis controlling for Binet stage (A + B vs. C) and cytogenetic risk (favorable + intermediate vs. unfavorable) showed a trend toward the independent association of MALAT1 expression with TTFT in U-CLL (hazard ratio (HR) = 0.581, 95% CI = 0.337−1.002, *p* = 0.051).

**Figure 6:**
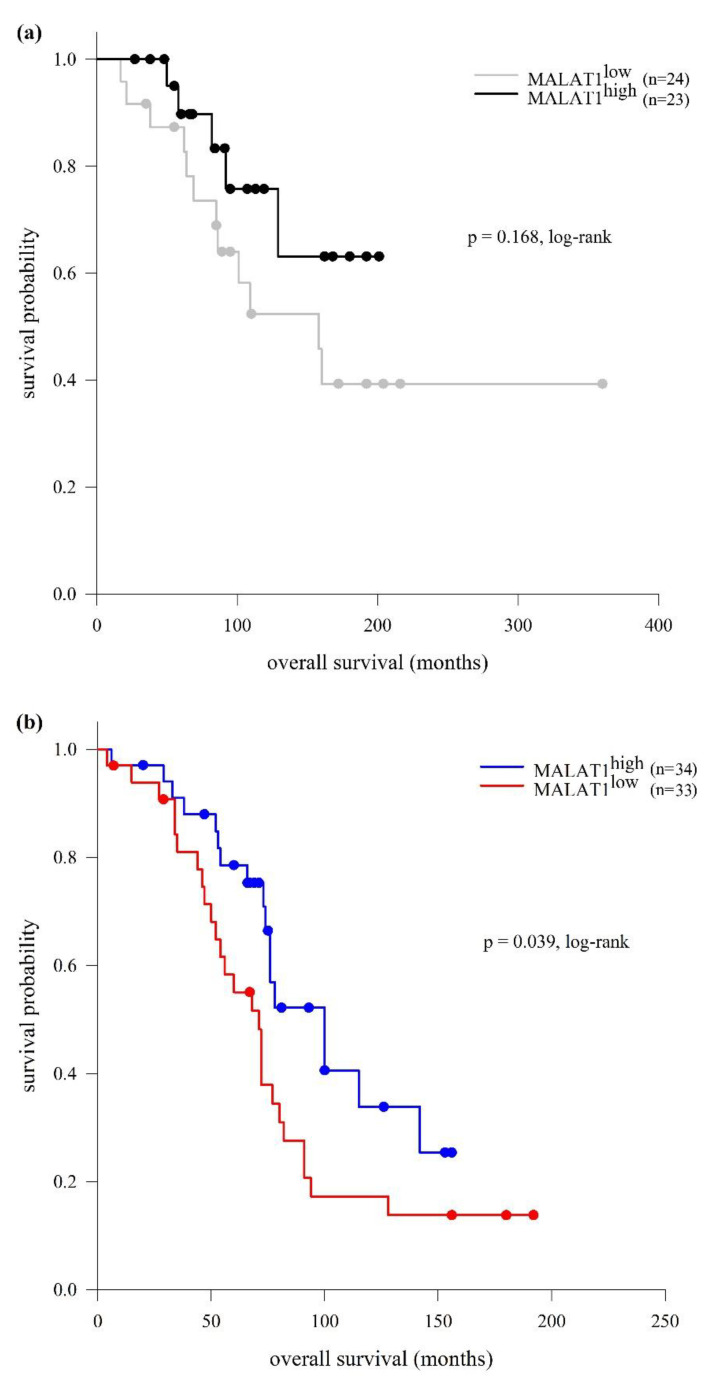
Kaplan–Meier analysis of overall survival (OS) in relation to MALAT1 expression in M-CLL (**a**) and U-CLL (**b**). Significant differences in OS between MALAT1^high^ and MALAT1^low^ group were observed in the U-CLL but not in the M-CLL subtype. (**a**) M-CLL (*n* = 47): median OS in MALAT1^high^ group was not reached and in MALAT1^low^ group was 158 months (95% CI = 87.249−228.751). (**b**) U-CLL (*n* = 67): median OS in MALAT1^high^ group was 100 months (95% CI = 69.244−130.756) and in MALAT1^low^ group 71 months (95% CI = 58.643−83.357).

Regarding OS in our CLL cohort, it was significantly longer in the MALAT1^high^ group in comparison to the MALAT1^low^ group (129 and 80 months, respectively; *p* = 0.029, log-rank test). As in the case of TTFT, in the M-CLL subtype, a difference in survival distributions between the MALAT1^high^ and MALAT1^low^ group was not observed (median OS not reached and 158 months, respectively; *p* = 0.168, log-rank test), while in the U-CLL subtype, median OS was significantly longer in the MALAT1^high^ vs. MALAT1^low^ group (100 vs. 71 months, respectively; *p* = 0.039, log-rank test) (Figure 6). However, multivariate Cox analysis controlling for Binet stage (A + B vs. C) and cytogenetic risk (favorable + intermediate vs. unfavorable) showed that MALAT1 expression was not an independent predictor of OS in U-CLL (HR = 0.625, 95% CI = 0.337−1.161, *p* = 0.137).

## 3. Discussion

By virtue of their capacity to modulate gene expression, lncRNAs are important regulators of both normal B-cell development and B-cell tumorigenesis. The aberrant expression of lncRNAs in B-cell neoplasms affects key signaling pathways associated with progression through cell cycle, proliferation, DNA damage response, apoptosis, survival, differentiation, and interactions of malignant cells with the microenvironment [40,41,42]. 

LncRNA MALAT1 has not been extensively investigated in mature B-cell malignancies, as opposed to other cancers. In a meta-analysis which encompassed 15 types of cancers including diffuse large B-cell lymphoma (DLBCL), multiple myeloma (MM), and CLL, high MALAT1 expression was found to be a good prognostic factor for OS in B-cell neoplasms [29]. However, other studies on separate disease entities yielded conflicting results regarding MALAT1 functions and prognostic significance, which were clearly dependent on the B-cell cancer type, as well as on the study design. 

In DLBCL patients and cell lines, MALAT1 was found to be upregulated, and its high expression was associated with good prognosis regarding OS [29]. However, MALAT1 knockdown in DLBCL cell lines induced cell cycle arrest, decreased proliferation and migration of tumor cells, and promoted apoptosis [21,43]. It was demonstrated that, by sponging miR-195, MALAT1 activates the expression of immune checkpoint molecule PD-L1, resulting in the proliferation, migration, and immune escape of DLBCL cells [21]. In addition, MALAT1 knockdown was shown to affect the expression of autophagy-related genes, leading to the activation of autophagy and reduction in chemotherapy resistance [43]. 

MALAT1 is also overexpressed in mantle cell lymphoma (MCL) patients and MCL cell lines, and its high expression has been associated with high and intermediate prognostic risk and poor OS. The knockdown of MALAT1 in MCL cell lines inhibited proliferation, induced apoptosis, and led to cell cycle arrest through the upregulation of cyclin-dependent kinase inhibitors p21 and p27. Mechanistically, MALAT1 silencing reduced the expression of EZH2, a component of polycomb repressive complex 2 (PRC2), and inhibited the recruitment of PRC2 to target genes p21 and p27, thus leading to their re-expression [44].

The upregulation of MALAT1 was reported in follicular lymphoma, and high MALAT1 levels were associated with shorter progression-free survival (PFS). Genes whose expression was found to be positively correlated with MALAT1 expression are involved in proliferation, migration, and angiogenesis, as well as in BCR and interleukin signaling [39].

In MM, Cho et al. reported the overexpression of MALAT1 in bone marrow mononuclear cells of newly diagnosed patients, but association with the percentage of plasma cells, PFS, and OS was not observed. The expression of MALAT1 was decreased after initial chemotherapy treatment, and a smaller decrease was associated with early progression [45]. However, in another study, high MALAT1 was associated with shorter OS and PFS, and its upregulation upon chemotherapy was linked to the extramodular spread of the disease [46]. On the other hand, in the study of Isin et al., plasma MALAT1 levels were lower in MM patients when compared to healthy controls and were associated with advanced clinical stage [47]. MALAT1 was shown to be associated with pathways involved in cell cycle progression, DNA damage response, proliferation, apoptosis, adhesion, and invasion, and multiple mechanisms of action in MM cells have been discovered [48,49,50,51,52]. Interestingly, increased MALAT1 expression was also observed in mesenchymal stem cells in MM, and it was demonstrated that MALAT1 enhances the transcription of the LTBP3 gene by recruiting the Sp1 transcription factor to its promoter. LTBP3 regulates the extracellular level of TGF-β, which is involved in the formation of bone lesions in MM [14].

The data on MALAT1 expression and prognostic relevance in CLL are scarce and, to the best of our knowledge, there are only two studies that have dealt with this topic. In both studies, the upregulation of MALAT1 in CLL was detected [38,39]. We investigated the expression pattern of MALAT1 in a cohort of 114 unselected CLL patients at diagnosis and performed a comprehensive analysis of its association with the diagnostic clinical, laboratory, and molecular characteristics of patients, as well as with the clinical outcomes.

The expression of MALAT1 in the peripheral blood mononuclear cells of our CLL patients was significantly higher than in the healthy controls, as it was found in previous studies [38,39]. MALAT1 levels were not associated with age at diagnosis, WBC, lymphocyte and platelet count, nor serum β2-microglobulin. In concordance with the finding of Fernandez-Garnacho et al., MALAT1 expression was not significantly different in M-CLL and U-CLL [39]. There was also no association of MALAT1 levels with BCR stereotypy in our cohort. However, we detected the association of high MALAT expression with several favorable prognostic factors, such as high hemoglobin, low serum LDH, and CD38-negative status. Furthermore, MALAT1 expression was higher in patients in earlier clinical stages (Binet A and Binet B) in comparison to patients in Binet C stage.

Previous studies reported the absence of an association of MALAT1 expression with chromosomal aberrations typical for CLL and the mutations in driver genes [38,39]. In our study, MALAT1 expression was not associated with *TP53* mutational status. In addition, the distribution of cytogenetic risk groups in MALAT1^high^ and MALAT1^low^ patients was not significantly different. However, when patients with favorable and intermediate cytogenetic risk were grouped together, we observed that they expressed lower MALAT1 levels in comparison to patients with unfavorable cytogenetic risk, defined by the presence of del11q22-23 and del17p13 which encompass ATM and TP53 genes, respectively. It has been reported that p53 directly binds to p53-binding motifs in the MALAT1 promoter and acts as a transcriptional repressor [53,54]. In fact, there is an inhibitory feedback loop in which MALAT1 regulates and is regulated by p53; MALAT1 represses p53 activity by inhibiting its transcription and acetylation, while p53 downregulates MALAT1 expression [53,55,56,57]. Moreover, MALAT1 expression has been reported to be negatively correlated to the expression of genes involved in DNA damage and repair pathways and *TP53*-mediated transcriptional regulation [39]. Thus, it can be speculated that the defects in the p53 pathway contributed, at least in part, to MALAT1 overexpression in patients with unfavorable cytogenetic risk that we observed, while in patients without del11q22-23 and del17p13, the upregulation of MALAT1 was a result of other mechanisms. The exact causes of MALAT1 dysregulation in CLL are still unknown. In the study of Fernandez-Garnacho et al., mutations and copy number alterations of MALAT1 locus were found to be rare and not associated with MALAT1 expression. In addition, MALAT1 levels were not related to the mutations of driver genes and DNA methylome profile, both of which have prognostic significance. However, the authors observed that MALAT1 expression was positively correlated to the expression of genes involved in pathways related to activation, proliferation, and survival of CLL cells in the lymph nodes (PI3K/AKT, MAPK, IL4, IL10), suggesting that microenvironmental stimuli may trigger MALAT1 upregulation [39].

We also investigated the association of MALAT1 expression with TTFT and OS. When analyzed in the whole cohort, MALAT1 expression status did not exert an association with TTFT. However, we observed a significantly longer TTFT in MALAT1^high^ in comparison to MALAT1^low^ cases in the U-CLL subtype (21 and 6 months, respectively) and a trend toward prognostic value independent of Binet stage and cytogenetic risk. It is noteworthy that there was no association of MALAT1 status with the CLL-IPI, which predicts TTFT in newly diagnosed CLL patients when the whole cohort was analyzed, as well as when M-CLL and U-CLL subtypes were analyzed separately [58]. Our results on TTFT contrast the findings of Fernandez-Garnacho et al. who reported the association of MALAT1^high^ status with shorter TTFT, which was evident in the whole cohort, as well as in the M-CLL, but not in the U-CLL subtype. The adverse impact of MALAT1 expression in M-CLL patients was also found when only patients in Binet A stage were analyzed. Furthermore, the authors did not observe the association of MALAT1 expression status with OS [39]. In our cohort, on the other hand, OS was found to be longer in MALAT1^high^ than in MALAT1^low^ patients (129 and 80 months, respectively) and, again, this association was also detected in U-CLL (100 and 71 months, respectively) but not in M-CLL. In the case of OS, the prognostic significance of MALAT1 expression was not independent of Binet stage and cytogenetic risk. The discrepancies between the results obtained in our study and the study by Fernandez-Garnacho et al. may be attributed to the differences in size and composition of the study cohorts, as well as to the methodological differences. Hence, further studies on larger, independent cohorts are warranted in order to clarify the impact of MALAT1 expression on CLL prognosis.

In conclusion, our results suggest that the upregulation of lncRNA MALAT1 may be involved in the pathogenesis and/or progression of CLL. However, there is not enough information about the functions of MALAT1 in CLL cells to explain the associations of MALAT1 expression with patients’ characteristics at diagnosis and the clinical outcomes that we observed. The factors causing its upregulation, as well as the regulatory networks in which MALAT1 participates in CLL B lymphocytes, have not been elucidated yet. Therefore, future research should focus on functional studies in order to investigate the mechanisms by which MALAT1 overexpression affects the pathobiology of CLL cells and whether these mechanisms differ between the M-CLL and the U-CLL subtype given their markedly disparate clinical behavior. It will also be important to investigate if the initially high MALAT1 expression changes during the disease course, as well as upon the administration of therapy, particularly novel targeted therapeutic agents. Deeper understanding of MALAT1 functional roles might bring it closer to becoming a valuable prognostic and/or predictive biomarker and, possibly, a therapeutic target in the management of CLL.

## 4. Materials and Methods

### 4.1. Patients and Samples

This retrospective study enrolled 114 unselected CLL patients who were diagnosed, treated, and followed at the Clinic for Hematology, University Clinical Center of Serbia (Belgrade, Serbia), and 20 healthy controls. The diagnosis of CLL was established according to the criteria of the International Workshop on Chronic Lymphocytic Leukemia (IwCLL) [59,60].

All the samples analyzed in this study were collected prior to the initiation of therapy. Standard demographic, clinical, and laboratory characteristics were determined at diagnosis, while molecular and cytogenetic analyses were performed either at diagnosis or during the period from diagnosis to the first treatment. The percentage of CLL cells and surface CD38 expression was determined using flow cytometry [61,62]. Cytogenetic abnormalities typically associated with CLL (del13q14, trisomy 12, del11q22-23, and del17p13) were detected by fluorescence in situ hybridization (FISH) on interphase nuclei obtained from peripheral blood using a panel of locus-specific probes (Vysis/Abbott Laboratories) according to the manufacturer’s instructions. *IGHV* SHM status and BCR stereotypy were analyzed as recommended by the European Research Initiative on CLL (ERIC) [32,63]. The *TP53* mutational status was determined using Sanger sequencing, as recommended by the ERIC [64]. Stratification of patients according to the CLL-IPI was performed as recommended by the International CLL-IPI Working Group [65].

### 4.2. Quantification of LncRNA MALAT1

The expression of MALAT1 was measured in peripheral blood mononuclear cells of CLL patients and healthy controls by qRT-PCR using TaqMan chemistry. Mononuclear cells were isolated from blood samples using Ficoll-Paque Plus (GE Healthcare, Chicago, IL, USA) density-gradient centrifugation. Total cellular RNA was extracted using TRI reagent (ThermoFisher Scientific, Waltham, MA, USA) and reverse-transcribed by RevertAid M-MuLV Reverse Transcriptase (ThermoFisher Scientific, Waltham, MA, USA) and random hexamer primers, according to the manufacturer’s instructions. qRT-PCR reactions were carried out in a final volume of 10 µL with 50 ng of cDNA using the TaqMan Gene Expression Assay for lncRNA MALAT1 (Hs03298492_g1) and TaqMan Universal PCR Master Mix (ThermoFisher Scientific, Waltham, MA, USA) in a 7900 HT Fast Real-Time PCR system (ThermoFisher Scientific, Waltham, MA, USA). The amplification of glyceraldehyde phosphate dehydrogenase (*GAPDH*) using the TaqMan Gene Expression Assay for *GAPDH* (Hs03298492_g1) served as the endogenous control. The cycling process was performed under default conditions recommended by the manufacturer: denaturation of the template at 95 °C for 10 min, followed by 40 cycles of 95 °C for 15 s and 60 °C for 1 min. All qRT-PCR reactions were run in duplicates.

Relative expression of MALAT1 was calculated using the comparative ddCt method, using healthy controls as the calibrator (ddCt = dCt_sample_ − dCt_healthy controls (median)_), and was expressed in terms of RU.

### 4.3. Statistical Analysis

Relationships between categorical variables, presented as absolute numbers and frequencies, were tested by χ^2^ or Fisher’s exact test. Continuous variables, presented using medians with ranges, 95% CI, and 25th and 75th percentiles, were analyzed using Student’s t-test and Mann–Whitney rank-sum test. The Shapiro–Wilk test was used to assess the data distribution. The correlation between continuous variables was analyzed using Spearman’s rank-order correlation test.

TTFT was defined as the time from diagnosis to the first line of therapy. OS was measured from the time of diagnosis to the time of death from any cause or the last follow-up. Survival analyses were conducted using the Kaplan–Meier method, and the differences in survival distributions between groups of patients were evaluated using the log-rank test. Cox regression analysis was performed to analyze the association between MALAT1 expression and TTFT and OS, and the results are presented as HR with 95% CI.

Statistical analysis was performed using SPSS 21.0 software (IBM, Armonk, NY, USA). For all analyses, *p* values were 2-tailed, and significance was defined as *p* < 0.05.

## Figures and Tables

**Table 1:** Clinico-biological characteristics of CLL patients in relation to lncRNA MALAT1 expression.

Variable	Global Cohort (*n* = 114)	MALAT1^low^ Group (*n* = 57)	MALAT1^high^ Group (*n* = 57)	*p*-Value
Sex (*n* = 114) ^a^, male/female	81/33	35/22	46/11	0.011 ^d^
Age (years)				
overall (*n* = 113) ^a^, median (range)	59 (33−80)	57 (33−80)	61.5 (38−76)	0.470 ^e^
˂65 (*n* = 76) ^a^, median (range)	56 (33−64)	56 (33−64)	55 (38−64)	0.676 ^d^
≥65 (*n* = 37) ^a^, median (range)	68 (65−80)	69 (66−80)	66 (65−76)	0.058 ^d^
WBC [×10^9^/L], (*n* = 99) ^a^, median (range)	37.6 (6.8−570)	34.1 (6.8−476)	44.4 (9.3−570)	0.264 ^d^
Ly [×10^9^/L], (*n* = 90) ^a^, median (range)	28.2 (2.7−558.6)	26.4 (2.7−447.4)	33.7 (5.6–558.6)	0.444 ^d^
Platelets [×10^9^/L], (*n* = 97) ^a^, median (range)	174 (1−432)	172 (1−432)	178 (3−361)	0.710 ^e^
Hb [g/L], (*n* = 98) ^a^, median (range)	139 (44−178)	132.5 (57−166)	143.5 (44−178)	0.004 ^d^
β2M [mg/L] (*n* = 59) ^a^, median (range)	3.2 (0.2−11.5)	3.3 (0.2−11.5)	3.2 (2−6.7)	0.974 ^d^
LDH level ^b^, (*n* = 77) ^a^, median (range)	1.4 (0.3−4.2)	1.5 (0.7−4.2)	1.1 (0.3−3)	0.004 ^d^
Binet stage (*n* = 113) ^a^, *n* (%)				0.250 ^f^
A	53 (46.9)	25 (44.6)	28 (49.1)	
B	44 (38.9)	20 (35.7)	24 (42.1)	
C	16 (14.2)	11 (19.6)	5 (8.8)	
CD38 status (*n* = 102) ^a^, *n* (%)				0.001 ^d^
positive (≥30%)	31 (30.4)	20 (40)	11 (21.2)	
negative (˂30%)	71 (69.6)	30 (58.8)	41 (78.8)	
Cytogenetic risk (*n* = 99) ^a^, *n* (%)				0.133 ^f^
favorable (del13q14) ^c^	32 (32.3)	18 (33.3)	14 (31.1)	
intermediate (no aberrations, trisomy 12)	43 (43.4)	27 (50)	16 (35.6)	
unfavorable (del11q22-23, del17p13)	24 (24.2)	9 (16.7)	15 (33.3)	
*IGHV* SHM status (*n* = 114) ^a^, *n* (%)				1.000 ^g^
mutated	47 (41.2)	24 (42.1)	23 (40.4)	
unmutated	67 (58.8)	33 (57.9)	34 (59.6)	
*TP53* mutational status (*n* = 61) ^a^, *n* (%)				0.536 ^d^
wt	50 (82)	31 (62)	6 (54.5)	
mutated	11 (18)	19 (38)	5 (45.5)	
CLL-IPI (*n* = 55) ^a^, *n* (%)				0.228 ^g^
low risk (score 0−1)	9 (16.4)	8 (21.1)	1 (5.9)	
intermediate risk (score 2−3)	18 (32.7)	14 (36.8)	4 (23.5)	
high risk (score 4−6)	23 (41.8)	13 (34.2)	10 (58.8)	
very high risk (score 7−10)	5 (9.1)	3 (7.9)	2 (11.8)	

WBC, white blood cells; Ly, lymphocytes; Hb, hemoglobin; β2M, β2-microglobulin; LDH, lactate dehydrogenase; *IGHV* SHM status, somatic hypermutational status of immunoglobulin heavy variable genes; wt, wild type; CLL-IPI, The International Prognostic Index for CLL; *n*, number of patients. ^a^ Number in brackets represents the total number of patients for whom the values of the given parameter at diagnosis/prior to the first therapy line were available. ^b^ LDH level was calculated for each patient as LDH [U/l]/upper limit of normal [U/l]; LDH level ≤ 1 was considered normal, and LDH level > 1 was considered elevated. ^c^ deletion 13q14 occurring as a sole chromosomal aberration. ^d^ Mann–Whitney rank-sum test. ^e^ *t*-test. ^f^ χ^2^ test. ^g^ Fisher exact test.

## Data Availability

The data that support the findings of this study are available from the corresponding author upon request.
